# The Impact of COVID-19 on Criminal Investigations and Proceedings in Sweden – A Snapshot of Practitioners’ Realities

**DOI:** 10.1016/j.fsisyn.2020.11.001

**Published:** 2020-11-06

**Authors:** Moa Lidén

**Affiliations:** Faculty of Law, Uppsala University, Sweden, Department of Security and Crime Science, UCL, United Kingdom

**Keywords:** COVID-19, Sweden, Crime

## Abstract

Since well-functioning criminal investigations and proceedings are crucial parts of a society governed by the rule of law, it is essential to understand whether and how they are impacted by the current COVID-19 pandemic. The Swedish context provides an interesting case study and comparison to developments in other countries which have introduced more far-reaching interventions such as lockdowns. In this study, Swedish practitioners (N =10), including police officers, forensic pathologists, prosecutors, defense counsels and judges, participated in an online survey in which they gave their views on the pandemic’s impact on their work situations. The overall results show that the major impact was on their work methods, with transitions to using more online methods and increased remote working, while the smallest impact was noted in relation to work character, including crime and evidence types which have remained relatively constant. Forensic pathologists reported the largest impact on work quality, although there was large individual variation in their responses. On a general level, the practitioners perceived that the measures undertaken at their work places were relatively sufficient and also perceived of the risk of contracting or spreading the virus as relatively low but also this was associated with large individual variation. Given the small sample of practitioners and that practitioners’ responses relate to an early stage of what is presumed to be a prolonged pandemic, replication as well as caution in interpreting the results are needed.

## 1 Introduction

Following the increased attention, concern as well as the World Health Organization’s (WHO) official declaration of the coronavirus outbreak as a global pandemic [1], much research has been conducted into its effects in a range of contexts, not the least the medical [[2], [3], [4]] and socio-economic [[5], [6], [7]]. However, the research into the legal context is still sparse and focuses on problems faced by specific categories of practitioners. For instance, it has concerned responses by police and prosecution to increases in crime rates and changes in crime types [[8], [9], [10]], difficulties that pathologist face in performing full medicolegal autopsies [11,12] and challenges with the transition to using more online work methods in Courts [13]. This research has had its bases in a range of jurisdictions but most of it focuses on the US, Canada or Italy. The Swedish legal setting provides an interesting case study since the Swedish state level response has not only been different from most other states, for example by refusing lockdown, but also been questioned on the basis of it being too mild to properly protect individuals and prevent further spreading of the virus [14,15]. Some media has even referred to the Swedish COVID-19 response as the “Swedish Covid Experiment” [16,17]. While today several other countries and states have started to reopen at least parts of their societies [18,19], it is still unknown whether differences in initial responses manifest themselves somehow, for example in the context of criminal investigations and proceedings. Swedish media reports that several police officers in the area of Gothenburg, all working within the same unit for gross crimes, have contracted the virus [20]. It is easy to see how such developments can greatly impair the ability to conduct efficient investigations, especially if the virus spreads further within the police force [21]. However, there are also more general media reports, for example about the case backlog arising in Swedish Courts as a consequence of the pandemic [22]. Such overall observations should be examined in more detail, confirmed or disconfirmed by practitioners, and compared to what is known about developments in other countries so far.

The purpose of this study is to examine how COVID-19, and preventive measures undertaken in response to it, impact on criminal investigations and proceedings in Sweden. While it is possible to examine this impact in many different ways, this study focuses on the perceptions of practitioners and more specifically police officers, forensic pathologists, prosecutors, defense counsels and judges. The views of these practitioners will also be compared to the findings regarding the impact of COVID-19 in other jurisdictions, to the extent that such impacts have been reported and published. To this end, a survey with Swedish practitioners was conducted and, through a literature search, their responses could, at least in part, be compared to findings relating to other jurisdictions. Since both the Swedish practitioners’ responses and the available reports from other jurisdictions concern a relatively early stage of a presumably prolonged pandemic, this enables a snapshot of practitioners’ realities rather than a complete report and comparison across jurisdictions throughout the course of the pandemic.

## 2 Theory

The literature search and review indicated that COVID-19, so far, has impacted practitioners’ work situations primarily in four different ways. These are: 1) the work methods, 2) the work load, 3) the work character and 4) the work quality, all of which are explained and exemplified below.

When it comes to work methods, reports from Courts in e.g. Canada and the US show that these Courts hold urgent trials via online technology while routine trials, jury trials and other proceedings have been cancelled or adjourned [13]. The Canadian approach to conducting trials using technology has been relatively cautious, especially in the upper instances, compared to the US [23]. For example, in Texas, an emergency order has been issued authorizing Courts to conduct criminal (as well as civil) proceedings through teleconferencing, videoconferencing or other means, with the exception of jury trials [24]. In these reports, it has been noted that delays and substantial case backlogs [25] may not only impact on the defendant’s constitutional rights, but also be a concern due to the rapid spread of COVID-19 as well as increased mental illness among detainees and prisoners [26,27]. As a consequence of the changes made in these Courts, the public and the media has more limited access to participate in the proceedings [13].

Also, the work load for practitioners in different countries seems to have been impacted. For example, work load has increased as a consequence of necessary adjustments due to changes in crime types [11]. However, in Italy, in less than 2 months, medico legal autopsies drastically decreased by 70% [28]. Also Canadian Courts have provided access only to limited essential legal services and most Courts are only hearing matters deemed urgent, like bail and release from custody cases while other cases are postponed or cancelled [13]. It is not clearly stated in the reports from Courts whether this has reduced their work load or just changed the character of their work, or possibly both.

Reports from different jurisdictions suggest that the incidence of certain types of crime or crime suspicions have changed, thus impacting on the work character of practitioners. For example, Italy has seen an increase in requests for help from victims of stalking and domestic violence [29] while criminal proceedings related to traffic and occupational deaths have decreased [11]. In the US, and more specifically South Carolina, increases in frauds (relating to COVID-19 economic impact payment) have been noted [30] as well as sexual harassment relating to housing issues [8]. Also, gun violence has increased, although with some variation between NY City, Chicago, LA and Baltimore [10,[31], [32], [33], [34]]. In Florida, NY and Massachusetts fewer vehicle related crashes and injuries have been noted as people minimize their driving [35]. Also, while not yet documented, Australian researchers anticipate that increases in illicit drug trafficking will occur [36].

There are also indications that work quality has been impacted. The available information in this regard points to autopsies as one possible problematic area. For instance, in Italy, full medicolegal autopsies are not being performed except for in extreme circumstances and more frequently, both in Italy and in Ontario, only targeted dissections are conducted [11,12,37]. This is due to the contraction risk entailed in conducting full autopsies. It is yet unknown whether this impacts materially on the cases in which pathologists are involved, but the risk in relation to work quality has been noted.

## 3 Method

### 3.1 Participants

Study participants were Swedish practitioners (N =10), 7 men and 3 women, in the following five categories; police officers (n=2), forensic pathologists (n=2), prosecutors (n=2), defense counsels (n=2) and judges (n=2). They were recruited through emails with invitations to participate. No compensation was offered for participation. Participants’ ages varied between 35 years and 57 years (*M* = 50.30, *SD* = 6.78) and the length of their experiences in their respective occupations ranged from 8 years to 28 years (*M* = 19.10, *SD* = 6.99).

## 4 Survey and survey themes

Participants were asked to complete an online survey, which had been created using the software Qualtrics. The survey contained questions relating to participant background (occupation, gender, age, years of experience) as well as questions on seven other themes. To get an overall picture of which measures have been undertaken at different workplaces, the first theme was “Measures undertaken at the work place to prevent the spread of COVID 19” (1). Thereafter, the themes identified through the literature review in section 2, that is, work methods, work load, work character and work quality (2–5) were introduced. Relating to the criticism regarding the relatively mild COVID-19 response in Sweden, practitioners were then asked about the risk they perceived of contracting or spreading COVID-19 through their work. Finally, to enable participants to share their experience regarding other possible impacts, also the theme “other impacts” (7) was included.

Any differences between different jurisdictions, between different categories of practitioners or between different individual practitioners in the same category can be both quantitative, in the sense that the impact is perceived as more or less pronounced, and qualitative, in the sense that the impact has manifested itself in different ways. This justifies the usage of both quantitative and qualitative measures in the survey. Hence, all survey themes, save for the last “other impacts”, had both quantitative and qualitative measures. These are described in more detail below.

**Theme** **1)** Measures undertaken to prevent the spread of COVID-19. Three questions were asked on this topic, a) “Please describe what measures have been undertaken at your work place or other places where you work to prevent the spread of COVID-19” (free text), b) “To what extent do you think the measures undertaken at your work place or other places where you work are sufficient to prevent the spread of COVID-19?” (scale 0–10, where 0 means “not at all”, 5 means “neither a little nor a lot” and 10 means “to a very large extent”) and c) “Please describe in what way you think the measures undertaken at your work place or other places where you work are sufficient or insufficient” (free text).

**Theme 2)** Impact on work methods. The questions posed on this theme were: a) To what extent has/does COVID-19 impact on your work methods (scale 0–10, where 0 means “not at all”, 5 means “neither a little nor a lot” and 10 means “to a very large extent”) and b) “Please explain how COVID-19 and the measures undertaken to prevent spread at your work place has/is impacting on your work methods”. To promote streamlining of participants’ responses the following explanatory note was included: “The term ‘work methods’ here refers to e.g. whether you have started working from home, need to respect social distancing rules, have meetings online or conduct other parts of your work online.”

**Theme 3)** Impact on work load. Under this theme, the following questions were posed: a) To what extent has/does COVID-19 impact your work load (scale 0–10, where 0 means “not at all”, 5 means “neither a little nor a lot” and 10 means “to a very large extent”) and b) “Please explain how COVID-19 and the measures undertaken to prevent spread at your work place has/is impacting on your work load”. The following explanatory note was added: “The ‘work load’ here refers to the number of hours you spend working each week”.

**Theme 4)** Impact on the work character. This theme consisted of the questions: a) To what extent has/does COVID-19 impact on the character of your work (scale 0–10, where 0 means “not at all”, 5 means “neither a little nor a lot” and 10 means “to a very large extent”) and b) “Please explain how COVID-19 and the measures undertaken to prevent spread at your work place has/is impacting on the character of your work”. The following explanatory note was added: “The term ‘work character’ here refers to e.g. whether you have noted differences in the crime (or crime suspicion) types or evidence types that you work with”.

**Theme 5)** Impact on the work quality. This included the questions: a) To what extent has/does COVID-19 impact on the quality of your work (scale 0–10, where 0 means “not at all”, 5 means “neither a little nor a lot” and 10 means “to a very large extent”) and b) “Please explain how COVID-19 and the measures undertaken to prevent spread at your work place has/is impacting on the quality of your work”. The following explanatory note was added: “The term ‘work quality’ here refers to factors like the balance between incoming and outgoing cases, processing times, objectivity, meticulousness and possibilities to interact adequately with involved individuals.”

**Theme 6)** Perceived risk of contracting or spreading COVID-19 through work. Under this topic, the following questions were posed: a) “To what extent do you fear contracting or spreading COVID-19 to others through your work?” (scale 0–10, where 0 means “not at all”, 5 means “neither a little nor a lot” and 10 means “to a very large extent”) and b) “Please explain in what way you fear contracting COVID 19 or spreading COVID 19 to others” (free text).

**Theme 7)** Other impacts of COVID-19. Under this theme participants were asked: “If you think COVID 19 has/is impacting on your work in other ways than those already addressed by the previous questions, please describe those other impacts here” (free text).

To ensure that the author correctly interpreted the responses provided by the participants, all participants had access to this paper before publication. The participants reported any deviations between how their responses were described in the paper and what they had intended when submitting their survey responses and the paper was updated accordingly.

## 5 Results and discussion

In the following, the survey results are presented and discussed in relation to available official statistics from Sweden as well as reports from other countries. Each category of practitioner is presented separately, and if there were differences between the responses from the two practitioners within the same category, these are pointed out.

Given the small sample size (N=10), quantitative measures are presented only in terms of descriptive statistics. Hence, quantitative differences do not represent statistically significant differences.

### 5.1 Measures undertaken to prevent the spread of COVID-19

The police officers both reported that social distancing is promoted in their work place, for example since lunch hours are separated into time slots to avoid overcrowding in the staff canteen. Also, appropriate Personal Protective Equipment (PPE), hand sanitizers and information are made available to all staff. Any staff member with symptoms is ordered to stay at home and only come back 48 h after being symptom free.

The forensic pathologists reported that new autopsy routines have been implemented, where all bodies are treated as potentially COVID-19 positive and the staff uses protective equipment such as particle filters and face shields or similar. For known COVID-19 cases, full autopsies might be replaced by external examinations so-called *likbesiktning,*^1^ depending on the strength of the *a priori* (before medico-legal examination) assumptions of cause of death and manner of death. Hence, before conducting autopsies, tests are being run on all bodies. Also, the necessary IT solutions have been provided and physical meetings replaced by videolink meetings.

The prosecutors reported that the major measures undertaken were recommendations to work from home. One of the prosecutors reported that if staff members show any symptoms they are prohibited to come to the office. Online meetings are used to a greater extent and, as far as possible, social distancing rules are applied in the office.

Among the defense counsels, one reported increased remote working, primarily among the administrative staff in the law firm and furthermore that TV-screens and software were installed to enable video link usage. The same defense counsel also reported that conferences and other events were cancelled and sanitary measures (e.g. placing hand sanitizers in the office) were undertaken. The other defense counsel reported that he/she is not remote working but still seeing clients face to face. In the contact with clients, he/she applies social distancing as far as possible, for example by avoiding to shake hands and also by suggesting phone meetings when possible. In the office where this defense counsel is working, hand sanitizers are available as well as information about COVID-19. In its communication with clients (summons to meetings and so on) the office instructs clients who have COVID-19 symptoms to refrain from attending the meetings in person.

Both of the judges, one Chief Judge in a District Court and one former District Court judge now working in a Court of Appeal, reported a shift towards conducting urgent Court hearings only or primarily. The urgent hearings entailed *inter alia* cases where legal deadlines apply, for example because the suspect is detained awaiting trial. Other hearings are cancelled or postponed. Both the prosecution and the defense are often offered to participate via videolink and this is usually accepted by the parties. One of the judges (Chief Judge in the District Court) reported that inside the Court room, social distancing rules are applied so that individuals in the audience are sitting only on every other chair. Also, lay judges over 70 years old are not taking part in the proceedings. Both of the judges reported increased remote working, online meetings and social distancing rules being applied among staff.

#### 5.1.1 Sufficiency of preventive measures

**Fig. 1:**
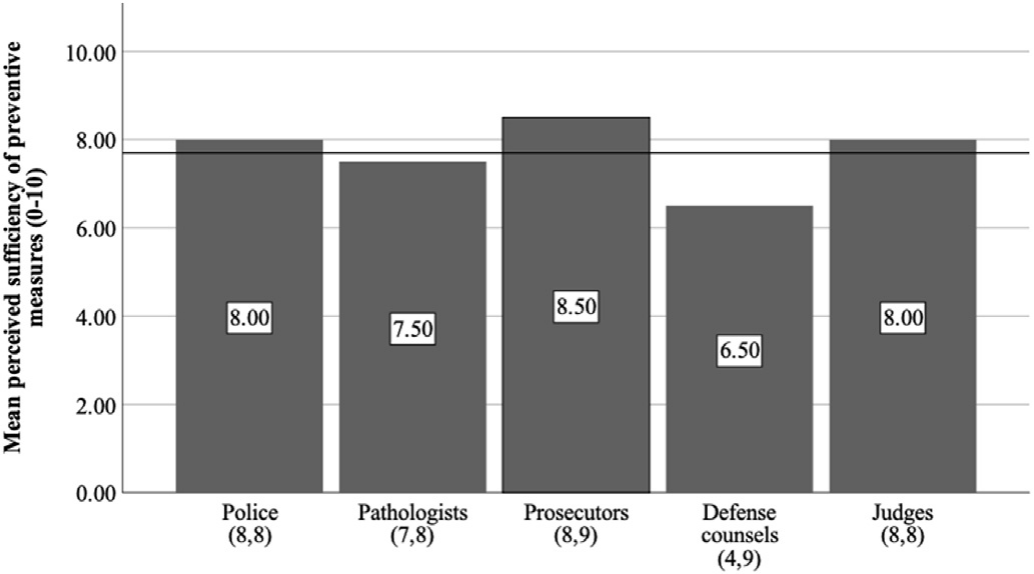
Practitioners’ perceptions of the sufficiency of measures undertaken to prevent the spread of COVID-19 in their work places or other places where they work. Mean across all categories of practitioners = 7.70.

As outlined in Fig. 1 below, most of the practitioners perceived of the measures undertaken as sufficient, with a mean rating of 7.70 (out of 10). Among the two defense counsels, the ratings varied the most, with the lowest rating being 4 and the highest rating being 9.

When asked to provide explanations of their ratings, one of the police officers, who had given a rating of 8, reported that the number of staff on sick leave was actually lower during the pandemic than before. Hence, this constitutes a difference compared to the reports in Swedish media according to which many police officers belonging to another geographical area (Gothenburg) had contracted the virus and were unable to work [20,21]. The other police officer explained his/her rating of sufficiency (also 8) differently, namely that there is a shortage of sanitizers for cleaning surfaces etc.

Similarly, both of the forensic pathologists stated the measures undertaken were reasonable given what is known about the infection risk, while one of them (rating 7) expressed difficulty with ensuring that locks (sluices) are functioning appropriately to separate clean and soiled areas in the ward.

Also the prosecutors believed that the measures undertaken were sufficient overall but one of them commented that it is difficult to maintain social distancing when appearing in person in Court.

The defense counsel with the highest rating (9) thought that the infection risk was sufficiently reduced through increased remote working and the social distancing applied in the office. However, the other defense counsel, who works in another office and another geographical area, with the lowest rating (4) expressed that only a few measures had been undertaken apart from social distancing as*: “we cannot stop seeing clients in person”*.

Both of the judges considered the undertaken measures sufficient and expressed that they had abided by the relevant recommendations from the authorities.

### 5.2 Impact on work methods

**Fig. 2:**
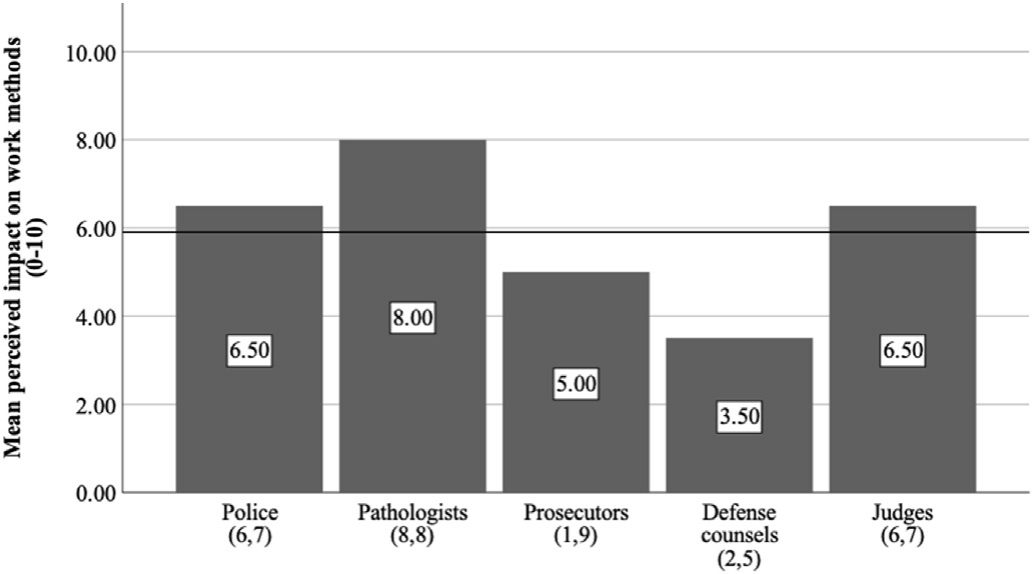
Perceived impact of COVID-19 and preventive measures on work methods. Mean across all categories of practitioners = 5.90.

Across all categories of practitioners increased remote working as well as online methods for meetings, briefings, presentation of cases/reports or Court participation were reported, although with some individual variation (primarily among the defense counsels), see Fig. 2 below. Hence, these results are similar to those found in the US and Canada, although it seems online methods are used to different extent in the Courts in these respective countries. There were also many examples of cancelled or postponed events (Court hearings, conferences, supplementary training etc.). One of the prosecutors had decided for him/herself to not work from home and one defense counsel could not work from home because of the necessity to see clients in person in his/her work setting. The Chief Judge from the District Court also reported that he/she faced some challenges with keeping the staff motivated and to maintain group cohesion.

### 5.3 Impact on work load

Among the police, one of the officers with managerial responsibilities reported an initial increase in his/her work load due to the need to plan and establish new routines. For the other police officer, the work load had remained more or less the same.

The pathologists reported that the case flow was slower due to the new autopsy routines and also that there was less administration, which is similar to the findings reported from Italy. One of the pathologists reported that he/she now spent more time explaining COVID-19 related issues/difficulties to the police.

The prosecutor who had been working from home experienced that he/she gets the same job done in fewer hours and that the digital meetings save time as one does not have to move physically from and within the work place. However, the prosecutor who did not remote work reported that the work load had been constant before and after the pandemic.

The defense counsels both reported a more or less constant work load. One of them explained that, despite being in the middle of a pandemic, he/she was still working with large cases in the District Court. The other defense counsel explained that for cases in which the suspects have been deprived of their liberty the work load was the same, while it was reduced for other cases due to cancelled Court hearings.

Similar to the police, the judge with managerial responsibilities (Chief Judge, District Court) reported an initial increase in work load due to the necessity to establish new routines. However, this went back to normal again as hearings were cancelled. The other judge reported that the total work load had been constant although there had been a shift in the sense that he/she works more with presentation of cases/reports and less with hearings. Notably, these developments are expected and also consistent with what has been reported from Canadian and American Courts where staff e.g. have to learn new technology and adjust to their new work situations.

**Fig. 3:**
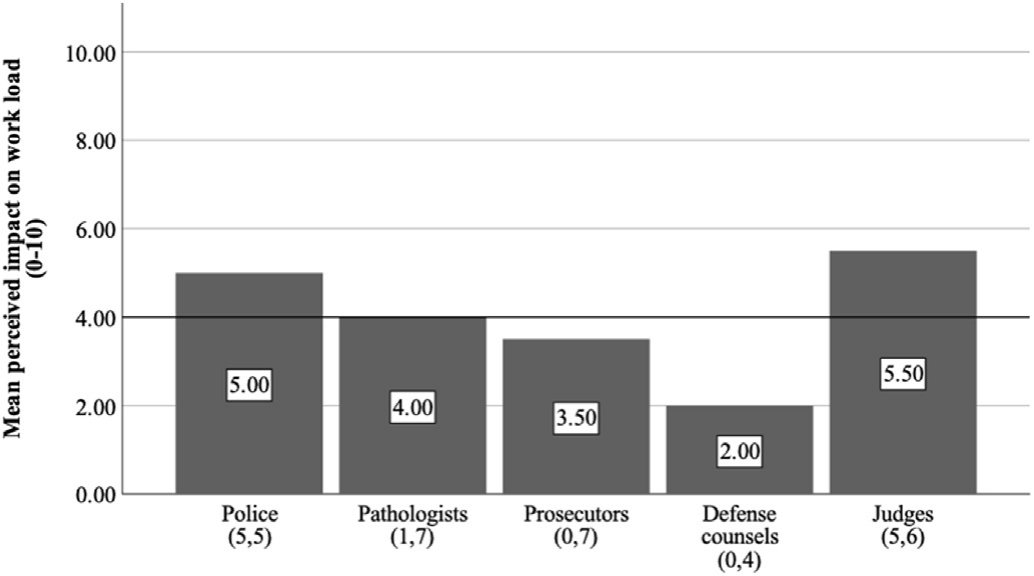
Perceived impact of COVID-19 and preventive measures on work load. Mean across all categories of practitioners = 4.00.

As illustrated in Fig. 3 below the overall perceived impact of COVID-19 on work load was fairly low, although with some variation across categories of practitioners as well as individual practitioners.

### 5.4 Impact on the work character

The police reported that the number of burglaries is reduced significantly since the outbreak of the virus. Hence, staff assigned to investigate burglaries can instead support investigations regarding e.g. vehicles. However, in relation to gross crimes, the police reported that the numbers from May 2020 are not any different than those from 2019. One of the police officers reported that more serial crimes were investigated, following a somewhat reduced work load.

None of the pathologists, prosecutors or defense counsels reported any changes in the type of crime suspicions, evidence etc. that they were working with. Neither the judges reported such differences but both of them explained that with the pandemic more preparatory was conducted instead of hearings. Also, the judges reported that more decisions are made in cases which can be decided on the basis of documents alone.

**Fig. 4:**
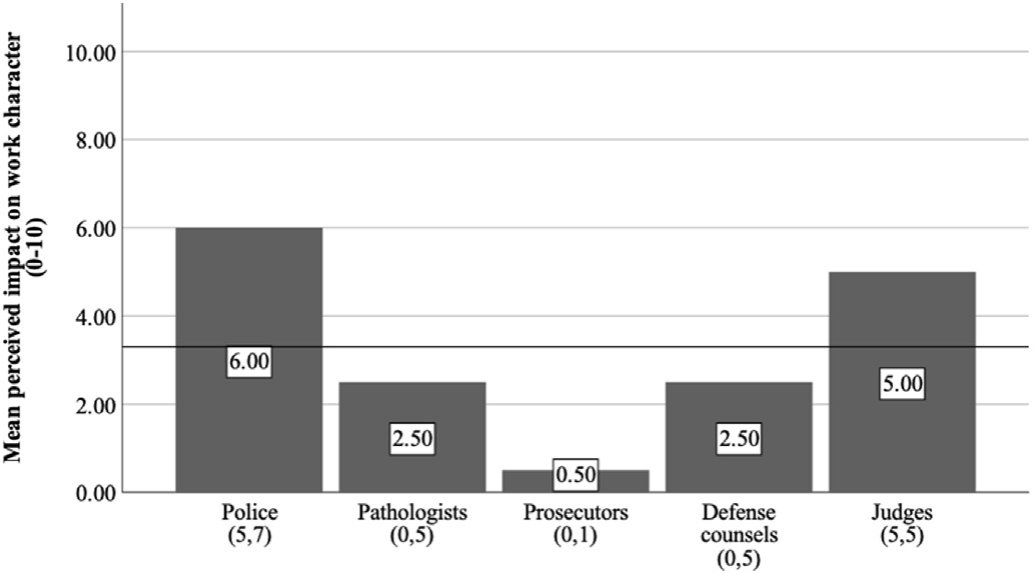
Perceived impact of COVID-19 and preventive measures on the work character. Mean across all categories of practitioners = 3.30.

As outlined in Fig. 4, the police and the judges reported the largest impacts on work character.

The responses provided by the practitioners can also be compared to the official statistics from The Swedish Police Authority concerning the incidence of reported crimes nationwide [38]. These statistics entail comparisons between crimes reported in March-April 2019 and in March-April 2020. The largest decrease was in relation to crimes of theft (−18.20%) while the largest increase was in relation to narcotic drug offences (+13.10%). Violent crimes against individuals had decreased (−8.40%).

Although decreases and increases in reported crimes do not necessarily mean that the actual incidence of crimes has changed, these statistics, as well as what is reported by Swedish practitioners seem to differ from what is reported so far from Italy, the US and Canada. In these countries there are not (yet) any reports of decreases in burglaries but instead increases have been noted in relation to gross crimes including gun violence and assault. It is unknown whether these noted differences are real and constant over time, or instead products of the limited information currently available.

### 5.5 Impact on the work quality

One of the police officers explained that since their work is conducted on the basis of best practice and guidelines, the work quality has not been impacted by COVID-19. The other police officer thought this question was difficult to answer but expressed a concern that the processing times at the National Forensic Centre (NFC) are longer than before.

One of the pathologists stated that *“there is a risk of missing important information when full autopsies are replaced by external examinations.”* He/she added that crime scene investigations may be complicated due to the usage of Personal Protective Equipment (PPE), since it results in reduced hearing, smell and sight. However, the other pathologist believed that the final products were better since more time could be spent on each case.

One of the prosecutors believed that digital meetings cannot fully replace ordinary real-life meetings when it comes to exchange of information and collaboration. The chances to interact and consult with colleagues more informally are reduced, which to a certain extent can impact on the work quality. The other prosecutor saw no differences in work quality.

One of the defense counsels noted that the quality of phone meetings was sometimes impeded due to technical issues. Also, this defense counsel noted that when the Courts conduct witness interviews via phone, this may have an impact on the judges’ assessments on witness credibility and reliability. The view of this defense counsel is in line with current day research suggesting that the format in which witnesses’ as well as plaintiffs’ testimonies are presented significantly influences perceptions of them and their testimonies [39,40]. For example, live testimonies are often considered more detailed and reliable than those presented via video or audio recordings [39,40]. It is unknown what the effect will be of the more general shift towards using technology rather than in Court testimonies following the pandemic. The other defense counsel believed the work quality was more or less constant.

One of the judges noted that the balance between incoming and outgoing cases has been impacted since many hearings have been cancelled. This has resulted in that the processing times are longer. The other judge stated that since the Court is prioritizing some cases above others the balances and processing times are worse for some types of cases but better for other types of cases. However, he/she did not think there were any other impacts on quality. These findings are similar to the backlogs reported in American and Canadian courts.

**Fig. 5:**
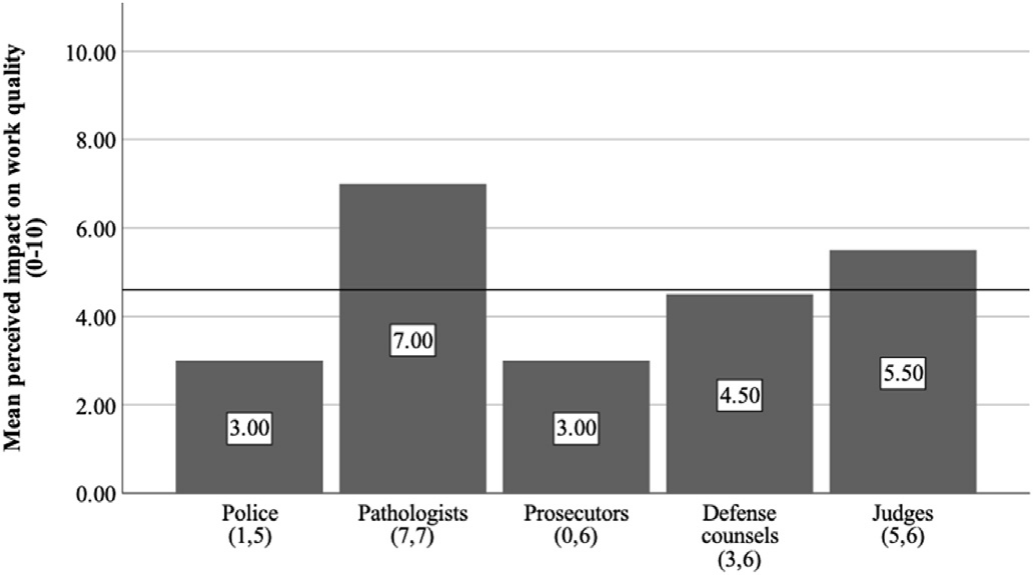
Perceived impact of COVID-19 and preventive measures on the work quality. Mean across all categories of practitioners = 4.60.

As illustrated in Fig. 5, pathologists reported the largest impact on work quality. However, as noted in their responses above, one of them perceived of this impact as negative and the other one as positive.

### 5.6 Perceived risk of contracting or spreading COVID-19 through work

One of the police officers reported practically no fear of contracting or spread the virus since he/she works with the same people every day and due to the preventive measures undertaken in their work place. The other police officer was mostly concerned about travelling to work because of an increase in the number of people using public transport. He/she stated that: *“I know I take responsibility and stay at home even* *at* *the slightest sign of being ill, but I am not sure everyone else is doing the same.”*

One of the pathologists explained that *“autopsy work entails an obvious risk to be exposed to aerosols from body fluids”*. He/she explained that, in fact, a number of his/her colleagues have contracted COVID-19, although it is always difficult to know exactly when, where and how they contracted it. The other pathologists did not fear contracting the virus, since he/she believed the exposure to COVID-19 is small and under controlled circumstances.

One of the prosecutors stated that since the virus can be transmitted also by non-symptomatic individuals *“the risk is everywhere and not larger in relation to the work place than other places, such as the grocery store.”* The other prosecutor did not feel particularly concerned to contract or spread the disease.

One of the defense counsels stated that he/she was exposed to many different individuals, for instance in small Court rooms, visits at detention centers and other contacts with clients. He/she added that a colleague had to travel by plane on repeated occasions within Sweden in order to attend a Court hearing. The other defense counsel perceived of the risk as pretty low since he/she avoids the work place and it has been empty for a long time period.

In the assessment of one of the judges, the largest risk is when travelling by train to the Court which he/she does practically every day, particularly since the trains are becoming more and more crowded, following an initial period when most people were working from home. The other judge reported that: *“Even though we abide by social distancing, a day at work entails quite a few meetings, but I am rarely very worried.”*

**Fig. 6:**
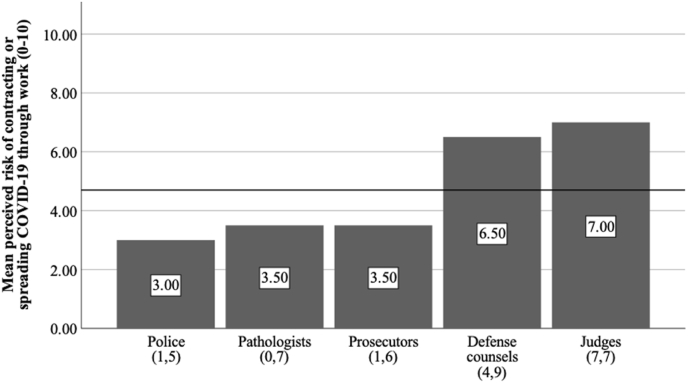
Perceived risk of contracting or spreading COVID 19 through work. Mean across all categories of practitioners = 4.70.

As outlined in Fig. 6, the judges and the defense counsels reported higher perceived risks of contracting or spreading COVID-19 than the police, pathologists and prosecutors, although this should be read in conjunction with the explanations above.

While there is large individual variation in the pathologists’ ratings (0,7), it can be questioned how a pathologist who directly interacts with a potentially or known COVID-19 positive body perceives of the risk as lower than a judge sitting in a Court room or a defense counsel interacting with his/her client. The more specific reasons for this are unknown but one possibility is that pathologists have access to and experience in using Personal Protective Equipment (PPE) and are also expected to use it, unlike judges and defense counsels who are left to social distancing measures which may appear more uncertain in effect and/or more difficult to apply in practice.

### 5.7 Other impacts of COVID-19

One of the police officers indicated some positive impacts of COVID-19 such as improving the preparedness to organize quick operative online meetings. He/she stated that it is likely they will continue working with such meetings also after the pandemic is over. Furthermore, the police stated that there are some remaining questions e.g. how to deal with items and bodies in safe ways. Not having any specific answers to these questions yet has been a cause of concern among some of the staff members.

One of the pathologists explained that they have been asked to answer some more general questions like whether elderly homes have undertaken adequate preventive measures in relation to COVID-19. According to this pathologist, such questions are impossible for them to answer.

One of the prosecutors added that sometimes witnesses do not want to come to Court due to the infection risk, which is far from ideal.

One of the defense counsels stated the inflow of cases to the firm was poor initially but that it is back to normal now.

One of the judges explained that some of the co-workers have been quite worried, and as their boss he/she has tried to deal with it appropriately. The worries have been general, in relation to the society, but also following new routines and instructions. Some co-workers have had issues working from home, as they feel stressed, ineffective or isolated. The other judge explained that in his/her opinion all the measures undertaken should be evaluated as to whether they are really necessary.

## 6 Conclusions

A snapshot is by definition a depiction of matter of states at a specific time and place and while this depiction may be accurate and informative of that specific time and place it is also limited to them. This research is limited to the experiences and perceptions of 10 Swedish legal actors, at what is presumed to be an early stage of the COVID-19 pandemic. It is therefore clear that the implications of this research may be limited both in time and in numbers, that is, it is unknown whether and to what extent the responses provided by these practitioners are representative of their respective occupational groups. Also, given that there are limits to the information available from other jurisdictions, comparisons can only be preliminary. For more nuanced and full comparisons between countries, it would be beneficial to use the same survey with practitioners also from other jurisdictions than the Swedish. However, this research has enabled some tentative conclusions, relating to 1) Between country comparisons, 2) Between category of practitioner comparisons and 3) Between individuals within the same category of practitioner.

As commented on in the Results and Discussion section, one possible difference between the Swedish and other jurisdictions is the impact that COVID-19 has had on the incidence of crime and crime types. According to the Swedish practitioners (the police) included in this study as well as official statistics, the number of reported burglaries and crimes of theft have decreased while for example reported narcotic drug offences and violent crimes against individuals have decreased. However, in countries like the US and Italy, the available reports point to increases when it comes to gun violence, vehicle incidents and assaults, suggesting, possibly, another type of pattern. Intuitively, it seems reasonable to expect that the number of burglaries entailing breaking in to someone’s home have decreased in all countries where people have spent more time in their homes during lockdowns. However, no reports are available in this regard. With the limitations that come from the available data so far it is uncertain whether this represents true and stable changes and differences but these possibilities should certainly be followed up and examined more systematically and on a larger scale in future research. Also, it is noteworthy that very little information is available so far regarding the incidence of domestic abuse following COVID-19. Indeed, the media as well as state authorities have expressed concern in this regard. For example, in the UK, the Government has provided official guidance to victims of domestic abuse on how to get help during the pandemic [41] and media campaigns have been launched encouraging the public to report any suspicions of domestic abuse [42]. It remains to be seen whether and to what extent the incidence of domestic abuse has changed and whether this may be different in countries where lockdowns have been implemented and in Sweden where no lockdown has taken place so far.

A similarity between what the Swedish pathologists report and what is reported from Italy, is that full autopsies are more rarely conducted (for known COVID-19 cases in Sweden) and this, potentially, entails a risk in relation to the quality of the pathologists’ work, for example because important information may be overlooked with more limited examinations. In the long run, it is possible that new methods will have to be developed to enable full autopsies and the potential of training as well as technology such as Virtual Autopsy Tables [43,44] and potentially robotic autopsies, inspired by robotic surgeries [45,46] may need to be evaluated by experts in the field.

Another partial similarity between Sweden and other countries is found in the Courts, since Swedish as well as American and Canadian judges report an increased usage of video technology. However, in the Swedish setting, it seems audiences as well as parties are allowed in the Court room to a larger degree, while maintaining social distancing.

The results in this specific study also enables some tentative comparisons across different categories of practitioners. Across all categories, the largest impact was reported in relation to work methods (*M*=5.90) and the smallest impact in relation to work character (*M*=3.30). To further evaluate and understand what transitions to online methods or other technological solutions mean seems relevant, for example when it comes to how judges evaluate reliability and credibility of witnesses, plaintiffs and others who, following the pandemic, participate via video link or phone on a more regular basis than before.

On a general level, across the different categories, the practitioners perceived that the preventive measures were relatively sufficient (*M*=7.70) and also perceived of the risk of contracting or spreading the virus as relatively low (*M*= 4.70) although with some variation between different categories as well as individuals, as exemplified by for instance differences in perceived risk which was higher among judges (7,7) than pathologists (0,7) but with large individual variation.

Noted differences between practitioners within the same occupational groups can be products both of differences in their more specific work situations and in their personalities. For example, there were individual differences in the pathologists’ perceived risk of contracting or spreading COVID-19 (0,7) and similar differences were noted between defense counsels (4,9), prosecutors (1,6) and police (1,5). Possibly, this is an indication that the perception of the impact/risk in relation to COVID-19 varies more between different individuals than it does between different categories of practitioners. There is also the possibility that the practitioners understood and applied the scales differently. Yet, the possible importance of specific work settings and personality may need further examination.

In sum, the major impact of COVID-19 on Swedish criminal investigations and proceedings is on the work methods of practitioners, most obviously with the transitions to more remote working and usages of online methods. All categories of practitioners have adjusted their work methods in some way, ranging from not shaking hands with clients to treating every single body as potentially COVID-19 positive during autopsy. The impact of COVID-19 on practitioners’ work methods is unsurprising and follows the developments reported from other jurisdictions. One potential area which deserves more attention is whether there are real and constant differences in the pandemic’s impact across different jurisdictions when it comes to the incidence of crime and increases/decreases in certain crime types. This research alone does not enable any conclusions as to whether the “Swedish Covid Experiment” [16,17] has had any significant impacts in this regard, but can possibly inspire more research into such questions. To enable more systematic examinations of this kind, practitioners should, ideally, be evaluated using the same or similar surveys and the survey used in this research can be used directly or further developed for such purposes. Also, practitioners’ reports should be supplemented with for example crime statistics and other data to study the impact of COVID-19 in a more holistic way. As a matter of work place policy and in the interest of the well-being of practitioners, it is relevant to further understand how their specific work situations and personalities impact on their ability to adjust effectively to new work conditions, especially given the presumably prolonged nature of the pandemic.

## Footnotes

1 Authors own addition: According to the Swedish Law (1995:832) about autopsies etc. § 4, a full autopsy entail opening of the body whereas ‘likbesiktning’ (Swedish) only entail external examination, blood testing and smaller interventions.
